# Association of 4% Articaine with Profound Inferior Alveolar Nerve Block Success in Third Molar Surgery Performed by Dental Students: A Three-Anesthetic Observational Study

**DOI:** 10.3390/dj14030183

**Published:** 2026-03-19

**Authors:** Thanyaphat Engboonmeskul, Rudjit Tunthasen, Kannika Rungsaeng, Panuwat Rassaiyakarn, Poonnapha Tanyacharoen, Panuwat Earkun, Teerawat Sukpaita

**Affiliations:** Department of Oral Surgery, Faculty of Dentistry, Naresuan University, Phitsanulok 65000, Thailand; thanyaphate@nu.ac.th (T.E.); rudjitt@nu.ac.th (R.T.); kannikar@nu.ac.th (K.R.); panuwatr62@nu.ac.th (P.R.); poonnaphat62@nu.ac.th (P.T.); panuwate62@nu.ac.th (P.E.)

**Keywords:** local anesthesia, articaine, undergraduate student, third molar surgery, dental education

## Abstract

**Background/Objectives**: An effective inferior alveolar nerve block (IANB) is critical for mandibular third molar surgery, especially for novice student operators who face steep learning curves. This study compared the clinical efficacy and safety of 4% articaine, 2% lidocaine, and 2% mepivacaine in an undergraduate setting. **Methods**: A prospective observational study was conducted with 189 patients undergoing third molar surgery performed by dental students. Patients received either 4% articaine (*n* = 69), 2% lidocaine (*n* = 61), or 2% mepivacaine (*n* = 59). Anesthetic efficacy was evaluated using a two-stage assessment comprising Vincent’s sign (Stage 1) and profound surgical anesthesia (Stage 2). Intra- and postoperative pain, anesthetic volume, surgical duration, and postoperative complications were recorded and compared among anesthetic groups. **Results**: Baseline demographics, impaction patterns, and difficulty indices did not differ significantly among groups. Stage 2 profound success rate was significantly higher with articaine (76.8%) than with lidocaine (55.7%) and mepivacaine (61.0%) (*p* = 0.031). Articaine was also associated with a longer duration of anesthesia (261.7 vs. 164.6 and 192.6 min; *p* < 0.001), a lower total anesthetic volume (2.1 vs. 2.4 and 2.3 mL; *p* = 0.007), and significantly lower intraoperative pain scores (14.3 vs. 31.0 and 29.8 mm on the Heft–Parker VAS pain scale (HPS); *p* < 0.001). Postoperative pain through Day 7 and complication rates were comparable among anesthetics, with no serious adverse events reported. **Conclusions**: Within the limitations of this observational study, four percent articaine was associated with higher profound IANB success rates and lower intraoperative pain observed in this cohort. These observed associations with higher success and tissue diffusion properties may mitigate the impact of novice technical variability within this cohort.

## 1. Introduction

Mandibular third molar surgery requires effective inferior alveolar nerve block (IANB) anesthesia for pain control [1,2,3]. The current knowledge indicates that this method of administering local anesthesia has a failure rate of up to 35% due to the diverse physical characteristics of patients [4,5,6]. Undergraduate clinical training in lower third molar surgery is commonly undertaken during the penultimate or final year of dental school, at a stage when operator competence in anesthetic administration and surgical skills is still developing [7,8,9]. Undergraduate settings face heightened risks: residents report 14.6% complication rates (pain, trismus, dry socket) versus experienced surgeons. Novice operators may experience IANB failure rates up to two to three times higher than those of experienced clinicians, attributed to imprecise needle positioning and an increased likelihood of repeat injections and neurotoxicity [10,11,12]. Local anesthetics commonly used for third molar surgery—2% lidocaine, 2% mepivacaine, and 4% articaine—are often combined with vasoconstrictors to prolong anesthetic effects and improve hemostasis, both of which are critical for surgical efficacy [13,14]. Several studies have compared the efficacy of local anesthetic agents in various dental procedures [15,16]. Recent meta-analyses support the superiority of 4% articaine (1:100,000 epinephrine) over 2% lidocaine for third molar surgery, with higher success rates, reduced onset time, better intraoperative pain control, and prolonged duration [17], attributes compensating for novice technical variability. A 2024 randomized controlled trial reported a significantly lower need for supplemental injections with articaine compared with lidocaine in third molar extractions (*p* < 0.05) [18]. Nonetheless, gaps in knowledge persist concerning the optimal anesthetic agent for the mandibular third molar surgery in undergraduate clinics. However, randomized controlled trial (RCT) in this setting face significant logistical and ethical hurdles; the high number of participating students and the inherent lack of standardized surgical experience make strict variable control difficult to achieve without disrupting the educational process. Therefore, the objective of this prospective observational cohort study was to compare two-stage IANB success and efficacy among 4% articaine, 2% lidocaine, and 2% mepivacaine in an undergraduate oral surgery clinical setting.

## 2. Materials and Methods

### 2.1. Study Setting and Operators

This was a prospective observational cohort in the undergraduate oral surgery clinic, Department of Oral Surgery, Faculty of Dentistry, Naresuan University. The prospective observational design was selected following consultation with the Institutional Review Board (IRB). It was determined that a formal RCT would require artificial constraints on operator skill and student numbers, which would fail to reflect the real-world clinical environment and the natural learning curves of undergraduate students. Thus, an observational approach was adopted to preserve the ecological validity of the study and maintain standard clinical decision-making. The operators were fifth- and sixth-year dental students on clinical rotations, which all of the students had experience with third molar surgery fewer than ten cases. The difficulty level of the cases was determined to be appropriate for the pre-graduate level according to the department’s criteria (the slightly to moderately difficult Pederson difficulty index) [19]. This research was conducted in accordance with the Helsinki Declaration and was approved by the ethics committee of Naresuan University, Thailand (approval COA No. 306/2024; approval date: 19 September 2024). The study was retrospectively registered at the Thai Clinical Trials Registry (TCTR20260110004) on 10 January 2026.

Inclusion Criteria:

- Presence of at least one mandibular third molar requiring surgical extraction, with the diagnosis and surgical necessity confirmed by a supervising oral surgeon.
- Case difficulty met the criteria for undergraduate student management and was defined as slightly to moderately difficult according to the Pederson Difficulty Index (Class I–II ramus relationship; mesioangular, horizontal, or vertical impaction; Level A–B depth).
- Age 18–45 years.
- Provided written informed consent after reviewing the study information.

Exclusion Criteria:

- Presence of local pathology, including periodontitis, pulpitis, apical periodontitis, or pericoronitis.
- Preoperative pain at the surgical site.
- Use of analgesic medications within 2 weeks prior to surgery.
- Known allergy to ibuprofen or any local anesthetic agent.
- Behavioral or psychiatric conditions impairing pain assessment, or communication barriers.
- Inability to attend the Day 7 postoperative follow-up.

Discontinuation Criteria:

- Terminated from research due to supervising faculty intervention; required manual/physical assistance from the supervisor beyond verbal guidance to ensure patient safety or successful extraction.
- Intraoperative emergency threatened safety, or participant withdrew consent.

### 2.2. Sample Size Calculation

The sample size was determined a priori using G*Power software (version 3.1.9.7; Franz Faul, University of Kiel, Kiel, Germany). For a three-group chi-square comparison of Stage 2 profound IANB success, a minimum of 57 participants per group (total *N* = 171) was required to achieve 80% power (1-β = 0.80) at a significance level of α = 0.05. The final analysis included 189 patients (*n* = 69 for articaine, *n* = 61 for lidocaine, and *n* = 59 for mepivacaine). A post hoc analysis confirmed that this final sample size provided sufficient power to detect the observed significant difference in profound success rates (76.8% vs. 55.7% and 61.0%; *p* = 0.031).

### 2.3. Anesthetic Selection Process

The selection of the anesthetic agent for each surgical procedure followed a structured clinical decision-making protocol to ensure patient safety and educational integrity:

1. Standardized Knowledge Base: Prior to the commencement of the study, all participating dental students attended a mandatory lecture covering the pharmacological properties, indications, and clinical differences between the three anesthetic agents (4% articaine with 1:100,000 epinephrine, 2% lidocaine with 1:100,000 epinephrine, and 2% mepivacaine with 1:100,000 epinephrine). This ensured a consistent baseline of knowledge among all operators.
2. Student Proposal (Case Talk): On the day of surgery, during the pre-surgical case discussion, the student operator was responsible for proposing a specific anesthetic agent as part of their comprehensive treatment plan for the assigned patient.
3. Clinical Supervision and Approval: The supervising oral surgeon reviewed the student’s proposal. The suitability of the chosen agent was discussed, taking into account the patient’s medical history, the radiographic difficulty of the mandibular third molar, and the student’s surgical experience.
4. Final Decision: The final decision on the anesthetic agent was made and approved by the supervising professor. This ensured that the choice was clinically appropriate for the specific complexity of each case, prioritizing patient safety over rigid randomization.
5. Availability of Supply: To prevent logistical bias, the dental clinic ensured that all three types of anesthetic agents were consistently available in stock and accessible for selection throughout the entire study period.

### 2.4. Clinical Procedure Protocol

Following informed consent, patients received detailed study explanations. Patients were instructed to rate the pain experienced via the 170 mm line Heft–Parker VAS pain scale (HPS) [20]. Prior to the procedure, all participants were calibrated on the use of the HPS. They were explicitly instructed to notify the operator if they experienced any intraoperative pain. In accordance with a standardized protocol, supplemental anesthesia was administered only when patients reported pain exceeding the 36 mm threshold, ensuring that clinician intervention was directly triggered by patient-reported discomfort rather than operator discretion. All patients signed approval after Q&A, medical histories were reviewed for comorbidities, allergies, and third molar symptoms while confirming inclusion/exclusion criteria. Under close faculty supervision, 5th or 6th year students administered the initial 1.5 mL of the local anesthetic solution to anesthetize the inferior alveolar nerve and lingual nerve, and the 0.3 mL remaining solution was administered to anesthetize the long buccal nerve. The injection speed was performed at 2 mL/min (~100 s) using a standard syringe with a 27-gauge 30-mm-long needle (Disposable Dental Needles, J Morita, Irvine, CA, USA). The prepackaged 1.8 mL local anesthetics utilized in this study include:

1. 2% lidocaine hydrochloride with epinephrine 1:100,000 (Lignospan standard, Septodent, France);
2. 2% mepivacaine hydrochloride with epinephrine 1:100,000 (Scandonest 2%, Septodent, France);
3. 4% articaine hydrochloride with epinephrine 1:100,000 (Artinibsa 4%, Inibsa, Spain).

**Figure 1:**
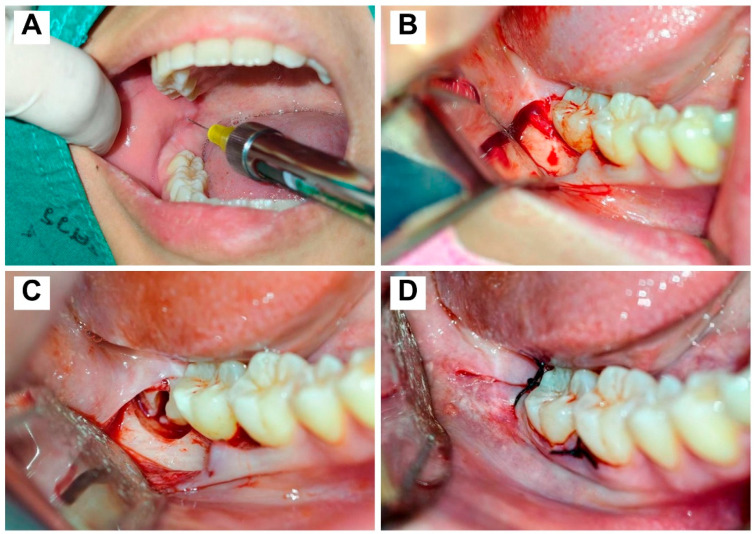
Undergraduate Mandibular Third Molar Surgery Protocol. Clinical photographs illustrating key steps of mandibular third molar extraction performed by 5th–6th year dental students under faculty supervision, including (**A**) injection of local anesthesia, (**B**) flap design, (**C**) bone removal/tooth sectioning, and (**D**) primary closure with 4–0 silk sutures.

Onset was assessed from injection start to subjective Vincent’s sign (lower lip numbness). After 10 min of full anesthesia verification (lip numbness + buccal mucosa), surgery proceeded if profound; otherwise, the same-anesthetic supplementation occurred: buccal deficiency via local infiltration of 0.3 mL, incomplete IANB via repeat of 1.2 mL (adjusted position), or intraoperative failure (tooth sectioning/elevation pain) via intraligamentary/intrapulpal injection of 0.6 mL. The sulcular full-thickness mucoperiosteal flap was elevated from the distal line angle of the lower first molar. Bone undercut removal or tooth splitting was done when necessary. The primary closure was performed using 4/0 silk suture. To mitigate operator-related bias and the inherent technical variability of the novice operators, all participating 5th- and 6th-year dental students underwent a standardized didactic and preclinical simulation workshop before the clinical rotation. Under faculty supervision, students were restricted to verbal guidance only; any case requiring physical faculty intervention (*n* = 24) was excluded from the final analysis to ensure that the reported success rates reflected purely student performance. Clinical photographs illustrating the basic procedure for mandibular third molar surgery by undergraduate students are shown in Figure 1.

**Figure 2:**
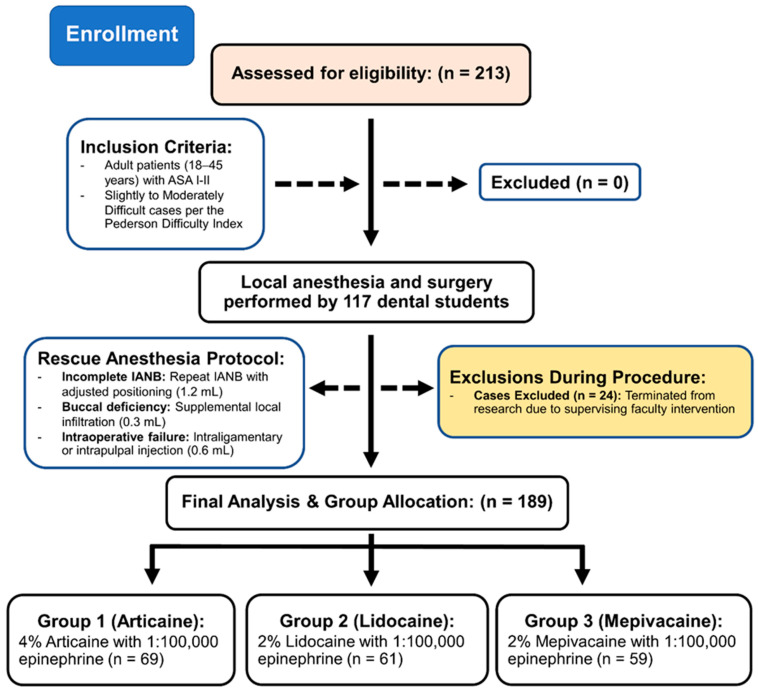
STROBE Flow Diagram of Study Cohort. Flow diagram illustrating patient recruitment, the standardized surgical and rescue anesthesia protocols, and the final cohort distribution after excluding cases with faculty intervention.

Intraoperative HPS was recorded immediately post-extraction, the participants placed a mark on the 170 mm scale where it best described their pain level. Total volume measured post-procedure via syringe markings. Standard surgical extractions followed with faculty oversight. Post-operatively, patients received ibuprofen 400 mg as needed (discontinue medication when HPS = 0). We administered an entire 500 mg amoxicillin regimen, including documentation of post-operative pain onset at 24 h (D1), 72 h (D3), and 168 h (D7). Day 7 suture removal assessed complications (infection, swelling, dry socket, trismus < 35 mm) with coded data entry. The study population specifically focused on undergraduate-performed third molar extractions under faculty supervision. To evaluate the potential impact of excluding cases requiring faculty physical intervention (*n* = 24), a sensitivity analysis was conducted by re-including these cases and classifying them as anesthetic failures. This study was conducted and reported in accordance with the Strengthening the Reporting of Observational Studies in Epidemiology (STROBE) Statement guidelines for prospective cohort studies [21]. Figure 2 shows the STROBE flow diagram representing participant recruitment and study progression. The chart details the standardized surgical protocol performed by 117 novice operators, the predefined rescue anesthesia steps, and the exclusion of 24 cases requiring faculty intervention to maintain the integrity of the novice-performance data. Final analysis was conducted on 189 cases across the three anesthetic cohorts.

### 2.5. Primary and Secondary Outcomes

The primary outcome was IANB success, categorized into two clinical levels:

1. Objective sensory blockade (Stage 1 success): Presence of Vincent’s sign and no pain upon sharp probing of the labial mucosa near the lower canine at 10 min post-injection.
2. Complete surgical anesthesia (Stage 2 success): Successful completion of the entire surgical procedure without the need for any supplemental anesthetic injections, which was defined as the ability to perform the surgery with no or weak pain (HPS rating ≤ 36 mm). This threshold follows the criteria of Gurucharan et al. (2022) [22], where ≤ 36 mm represents ‘none to weak pain’ signifying clinical efficacy sufficient for performing surgery without supplemental intervention.

Secondary outcomes obtained in this study include:

1. Onset (min): documented from the time of injection to the start of ipsilateral lip anesthetic as subjective symptoms.
2. Surgical duration (min): recorded from the time the incision was made until the last stitch was applied.
3. Anesthetic duration (min): the time from the onset of numbness to the complete return of sensation. Patients were provided with a printed home-entry diary and instructed to record the exact time their sensation returned. These records were collected at the follow-up appointment.
4. Total anesthetic volume (mL): the cumulative volume (mL) of all local anesthetic injections administered during the surgical procedure, including any supplemental injections.
5. Intra-operative pain (mm): the pain intensity during the surgical procedure, assessed immediately after surgery using the HPS.
6. Post-operative pain at D1, D3, and D7 (mm): the pain intensity assessed at 24, 72, and 168 h after surgery using the HPS.
7. Post-operative complications: the complications assessed on the seventh day after surgery, including infection, swelling, dry socket, and trismus (maximum mouth opening < 35 mm).

### 2.6. Statistical Analysis

The Statistical Package for the Social Sciences (SPSS) software version 26.0 (IBM Corp., Armonk, NY, USA) was used for analyzing the data. For continuous variables, we used descriptive statistics including means and standard deviations (SD). For categorical variables, we used frequencies and percentages. The Shapiro–Wilk test was utilized to verify when the continuous data distribution, such as the onset, duration, surgical time, and pain scores, was normal. The data met the criteria for parametric analysis; therefore, one-way analysis of variance (ANOVA), performed by Tukey’s post hoc test, was utilized to compare the mean values among the three anesthetic groups (articaine, lidocaine, and mepivacaine. The Pearson’s chi-square test was used for categorical outcomes, including the success rates of Vincent’s sign (Stage 1) and profound anesthesia (Stage 2), and the rates of postoperative complications. Fisher’s exact test was used when the expected number of cells was less than five. Pairwise comparisons between groups for categorical data were performed using chi-square tests with Bonferroni correction to identify specific differences. Primary outcome analysis utilized a mixed-effects logistic regression to adjust for confounding and operator-clustering. Anesthetic type, difficulty, and experience were included as fixed effects, while the individual operator was treated as a random effect. All statistical tests were two-tailed with a significance level of 0.05; however, *p*-values for secondary outcomes were considered exploratory and were not adjusted for multiple comparisons.

## 3. Results

### 3.1. Baseline Demographic Characteristics of the Patients

The primary endpoint of this study was Stage 2 anesthetic success (profound anesthesia), defined as the ability to complete the surgical procedure without supplemental injections and with intraoperative pain scores ≤ 36 mm on the HPS. Based on the inclusion criteria, a total of 189 mandibular third molar surgeries performed by 5th–6th year dental students were included in the final analysis. Participants were recruited between January 2025 and December 2025. Patient baseline characteristics, including age, sex distribution, impaction pattern, and Pederson difficulty index, were comparable among the articaine, lidocaine, and mepivacaine cohorts (Table 1). No significant inter-group differences were observed in demographic or radiographic baseline parameters (*p* > 0.05). The distribution of the 189 procedures among 117 operators is detailed in Supplementary Table S1. The vast majority of operators (95.7%) performed only 1 or 2 cases (mean 1.61 cases per student), ensuring the results represent novice clinical performance.

The 24 faculty-assisted cases were excluded as their interventions stemmed from surgical complexity rather than anesthetic failure (Supplementary Table S2). The primary reasons for intervention included failure in tooth sectioning/bone removal (*n* = 15), difficulty in elevating tooth fragments (*n* = 6), and unsuccessful flap reflection (*n* = 3). These cases were evenly distributed across groups (Articaine: 7, Lidocaine: 9, Mepivacaine: 8), minimizing the risk of attrition bias and ensuring the reported success rates accurately reflect student-led performance.

### 3.2. Anesthetic Efficacy and Success Rates

**Figure 3:**
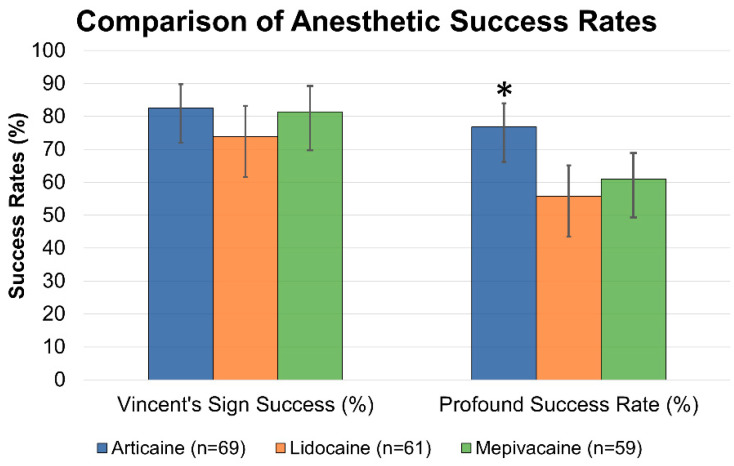
Comparison of Anesthetic Success Rates Among Three Local Anesthetics. Bar graph comparing Stage 1 objective sensory blockade (Vincent’s sign) and Stage 2 complete surgical anesthesia among articaine, lidocaine, and mepivacaine; Stage 2 profound success is significantly higher with articaine, whereas Stage 1 success is comparable across groups. Data are presented as percentages (%). Statistical significance was determined using Pearson’s chi-square test with Bonferroni correction for multiple pairwise comparisons (asterisk *p* < 0.05; SD error bars).

Regarding the primary outcome, Stage 1 objective sensory block (Vincent’s sign with negative sharp probing at 10 min) was achieved in a high proportion of cases across all three anesthetic agents, without a statistically significant difference between groups (*p* > 0.05). Stage 2 complete surgical anesthesia, defined as case completion without any supplemental injections, showed 4% articaine with 1:100,000 epinephrine achieved a significantly higher Profound Success Rate (Stage 2) at 76.8% (53/69), compared to 55.7% (34/61) for lidocaine and 61.0% (36/59) for mepivacaine (Figure 3). After adjusting for surgical difficulty and operator experience via mixed-effects regression, articaine remained significantly associated with higher Stage 2 success (Odds Ratios (OR) 2.41, 95% Confidence Intervals (CI) 1.24–4.68, *p* = 0.009). The Intraclass Correlation Coefficient (ICC) of 0.07 indicated minimal operator-level clustering (Supplementary Table S4). A sensitivity analysis including the 24 cases requiring faculty intervention (total *N* = 213), with all such cases treated as Stage 2 failures, yielded consistent results. Articaine maintained a significantly higher success rate (69.7%) compared to lidocaine (48.6%) and mepivacaine (53.7%) (OR 2.12, 95% CI 1.12–4.01, *p* = 0.021; Supplementary Table S5). Most failures in all cohorts were managed effectively with protocolized rescue strategies, including supplemental IANB, local infiltration, or intraligamentary injections, and did not necessitate case abandonment. A detailed breakdown of both primary and secondary outcomes, including the independent analysis of supplemental injection rates and intraoperative pain categories (≤36 mm vs. >36 mm), is provided in Supplementary Table S3.

### 3.3. Anesthesia Duration vs. Surgical Time and Pain Intensity

**Figure 4:**
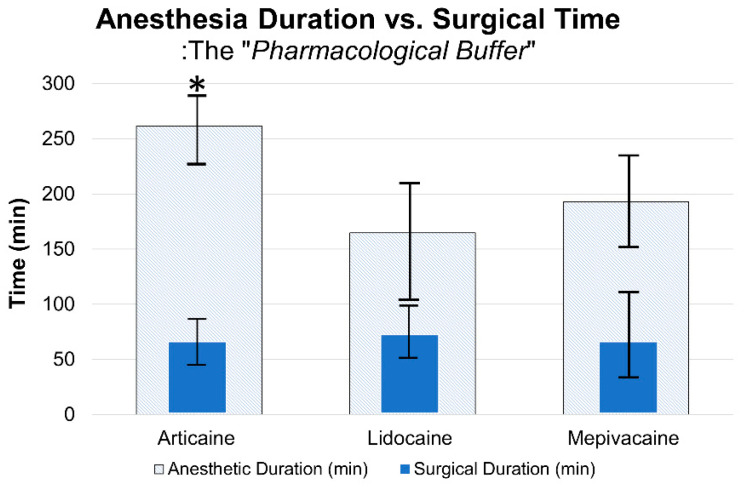
Anesthesia Duration vs. Surgical Time: The “Pharmacological Buffer”. A plot showing mean anesthesia duration and mean surgical time for each anesthetic; articaine provides a substantially longer pharmacological safety buffer than lidocaine and mepivacaine for prolonged student-led procedures. Statistical comparisons were performed using one-way ANOVA followed by Tukey’s post hoc test (asterisk *p* < 0.05; SD error bars).

Anesthesia duration was recorded via patient-maintained home diaries to capture the return of sensation in a real-time manner. Although this method is susceptible to potential recall bias, it provided the most practical means of longitudinal monitoring after discharge. The onset of anesthesia was similar across all groups, with mean times ranging from 2.1 to 2.2 min (*p* = 0.133). However, the duration of anesthesia was significantly longer for articaine (261.7 ± 34.8 min) compared to lidocaine (164.6 ± 48.3 min) and mepivacaine (192.6 ± 33.0 min) (*p* < 0.001). The mean surgical duration was 67.6 ± 21.8 min for articaine, 73.1 ± 23.1 min for lidocaine, and 78.1 ± 27.9 min for mepivacaine (*p* = 0.054). When comparing the “safety buffer” (anesthesia duration minus surgical time), articaine provided the most substantial window for instructional delays and faculty verification (Figure 4).

### 3.4. Total Anesthetic Volume and Intra-Operative Pain

**Figure 5:**
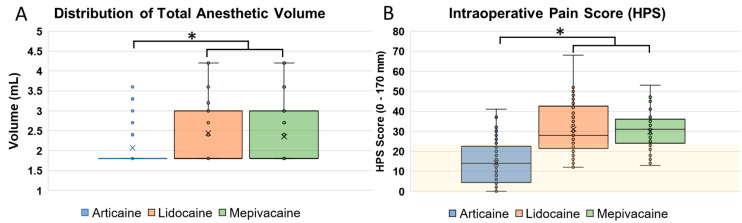
Distribution of Total Anesthetic Volume and Intraoperative Pain Scores. Box-and-whisker plots illustrating total anesthetic volume and intraoperative Heft–Parker pain scores; (**A**) articaine requires lower volume and (**B**) yields pain scores concentrated within the no-to-faint pain range (0–23 mm), with significant differences versus lidocaine and mepivacaine. The box plots represent the median, quartiles, and range of the data. Significant differences were identified using one-way ANOVA and Tukey’s post hoc test. (asterisk *p* < 0.05).

Cases receiving articaine showed significantly lower intraoperative pain scores with a lower volume of anesthetic. The total volume used in the articaine group (2.1 ± 0.5 mL) was significantly lower than in the lidocaine (2.4 ± 0.8 mL) and mepivacaine (2.3 ± 0.8 mL) groups (*p* = 0.007). Crucially, intra-operative pain scores (HPS) were significantly lower for articaine (14.3 ± 10.8) compared to lidocaine (31.0 ± 12.6) and mepivacaine (29.8 ± 8.8) (*p* < 0.001). The mean HPS scores in the articaine group were consistently maintained within the “no pain to faint pain” range (0–23 mm) throughout the procedure. Figure 5 shows box-and-whisker plots of the total anesthetic volume and intraoperative pain scores.

### 3.5. Post-Operative Pain and Complications

**Figure 6:**
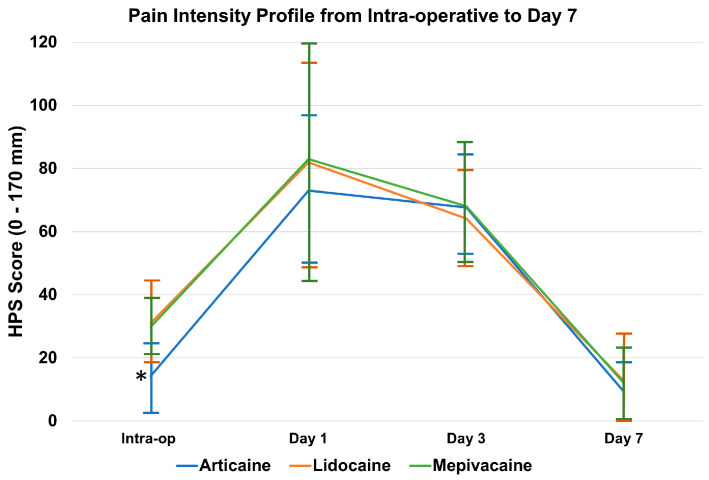
Pain intensity profile (HPS) from intra-operative to postoperative Day 7. Line graph showing mean pain scores measured by the Heft–Parker Visual Analog Scale (HPS). Error bars represent the Standard Deviation (SD). Statistical analysis at each time point was conducted using one-way ANOVA with Tukey’s post hoc test. Articaine demonstrated significantly lower intra-operative pain scores compared to other groups (asterisk *p* < 0.001). No significant differences were observed during the postoperative follow-up period.

The pain intensity profile from the intra-operative phase to postoperative Day 7 is illustrated in Figure 6. Postoperative pain assessed by HPS at Day 1, Day 3, and Day 7 followed the expected temporal decline in all groups, with the highest scores seen at 24 h and progressive reduction by Day 7. There were no statistically significant differences in postoperative pain trajectories among articaine, lidocaine, and mepivacaine at any timepoint (*p* > 0.05). Regarding postoperative morbidity, the incidence of complications was low and did not differ significantly between groups (*p* > 0.05). Dry socket occurred in 1.4%, 3.3%, and 1.7% of patients in the articaine, lidocaine, and mepivacaine groups, respectively. Jaw trismus was reported in 4.3% (articaine), 4.9% (lidocaine), and 5.1% (mepivacaine) of cases. No serious adverse events, including major neurological events, were observed within the 7-day follow-up period; however, the sample size (189 cases) and follow-up duration are insufficient to evaluate rare complications such as persistent paresthesia.

## 4. Discussion

This real-world observational study observed associations with higher profound anesthesia success rates (76.8% vs. 55.7–61.0%; *p* = 0.031) and lower intraoperative pain scores for 4% articaine in the context of mandibular third molar surgery performed by undergraduate dental students. Our findings indicate an association between 4% articaine and a higher rate of profound anesthesia, as well as lower intraoperative pain scores, despite a lower total volume used. The mean surgical duration in this study (approximately 67–78 min) is notably longer than durations reported for general practitioners or oral surgeons, which typically range from 15 to 30 min for similar difficulty levels [23,24,25].

The efficacy of IANB is a critical factor in the undergraduate oral surgery curriculum, where novice performance is often challenged by steep learning curves and procedural anxiety [8,26]. In this cohort, cases receiving 4% articaine showed higher Stage 2 success rates (76.8%), potentially facilitating focus on surgical techniques during the learning curve. This extended duration and the inherent technical challenges faced by students can be explained by the learning curve of third molar surgery. A prospective cohort study identified a critical proficiency milestone after the completion of approximately 10 cases, after which surgical time and complications significantly decrease. As the undergraduate students in this study were in their initial learning phase—often performing their first few clinical cases—they had not yet reached this stability milestone [27]. Beyond surgical dexterity, the educational protocol requires step-by-step verification by supervising faculty at critical stages (e.g., flap design, bone removal, tooth sectioning). Research by Sánchez-Torres et al. supports this, noting that surgical time is greatly prolonged when procedures are conducted by students, owing to both precise technique and the educational necessity for intraoperative evaluation [28]. In this “stop-and-go” environment, the prolonged duration places importance on the duration of action. The longer duration of 4% articaine was associated with a wider clinical window for students to complete the surgery without the stress of waning anesthesia, which often leads to higher intraoperative pain.

These observations align with prior studies reporting higher IANB success rates with articaine in student-performed procedures. Muhammad et al. reported that 4% articaine significantly improved IANB success rates for simple exodontia performed by students [29]. This advantage is particularly crucial for novice operators; articaine’s unique thiophene ring and higher lipid solubility are thought to contribute to its ability to penetrate dense cortical bone and lipid-rich nerve membranes [1,30,31]. As suggested by a previous study, these superior diffusion properties may potentially mitigate the challenges related to imprecise needle placement, a common technical error among students who may not yet have mastered the precise anatomical localization of the mandibular foramen [29]. Consequently, cases receiving articaine showed a higher profound success rate (76.8%) in our study, potentially reducing the need for stressful supplemental injections and being associated with higher operator confidence during the early stages of clinical training. While our primary analysis excluded cases requiring faculty intervention, the sensitivity analysis (Supplementary Table S5) suggests that the observed benefits of articaine are robust across different population definitions. Nonetheless, findings should be generalized specifically to student-completed extractions within a clinical training environment.

A key strength of this study is the two-stage assessment, distinguishing subjective nerve blockade (Stage 1) from surgical anesthesia (Stage 2). This reveals that while Stage 1 is often achieved, articaine showed a higher probability of reaching the pulpal and bone-cutting depth required for Stage 2. Despite its efficacy, the use of 4% articaine in IANB remains a subject of debate due to concerns regarding neurotoxicity and paresthesia. Retrospective studies, such as those by Haas and Lennon, suggested a higher incidence of nerve injury associated with 4% solutions [32]. However, this controversy has been largely mitigated by more recent prospective clinical trials and systematic reviews. Martin et al. and a large-scale meta-analysis by Nogueira et al. found no statistically significant difference in the incidence of permanent nerve damage between articaine and other amides [17,33]. The consensus in contemporary literature suggests that nerve injury is more likely related to mechanical trauma from the needle or the surgical procedure itself rather than the chemical properties of Articaine [34,35]. In our study, no cases of paresthesia were recorded within the 7-day observation window. While these results align with recent trials suggesting the safety of 4% articaine, it is important to note that the study’s power and follow-up duration were insufficient to draw definitive conclusions regarding rare long-term neurosensory complications.

It is important to consider that pain perception and anesthetic success are significantly influenced by psychological factors, particularly dental anxiety. Anxious patients often exhibit a lower pain threshold and higher autonomic arousal, which can lead to a perceived failure of the IANB [6]. While our study focused on anesthetic agents and student operators, the interplay between the patient’s psychological state and the ‘profoundness’ of anesthesia remains a critical variable that should be explored in future research to enhance clinical success.

This study has several limitations. First, its observational, non-randomized design limits direct causal inferences. Selection bias and confounding by indication may occur. Although baseline characteristics were comparable across groups, unmeasured confounders (e.g., case-specific factors influencing clinician anesthetic selection during pre-surgical case discussion and supervisor approval) cannot be ruled out and may contribute to the observed differences. The clinical recommendations should be viewed as provisional. Second, although mixed-effects modeling was employed to account for operator clustering (ICC = 0.07, Supplementary Table S4), further investigation in larger cohorts may provide additional precision for multilevel effects. Third, subjective pain perception may be influenced by unmeasured dental anxiety, which is known to significantly compromise IANB success. The lack of anxiety screening remains a factor that could impact the perceived depth of anesthesia. Fourth, while postoperative pain levels were monitored, the precise quantity of analgesic medication consumed by participants was not recorded. We acknowledge that variations in analgesic intake between groups may have influenced the postoperative pain comparisons. Future studies should include a standardized analgesic diary to more accurately account for this potential confounding factor. Lastly, the seven-day follow-up was insufficient to detect rare, long-term neurosensory complications. Future studies should utilize a double-blind, RCT design and standardize operator experience to further minimize potential biases.

## 5. Conclusions

Within the limitations of this observational study, the tested formulation of 4% articaine demonstrated favorable clinical efficacy compared to 2% lidocaine and 2% mepivacaine for mandibular third molar surgery performed by undergraduate dental students. In this study, this specific articaine preparation was associated with a significantly higher rate of profound anesthesia success and lower intraoperative pain scores, even with a lower total volume of anesthetic solution. While all three agents showed similar postoperative pain profiles and complication rates during the study period, observations within this cohort suggest that 4% articaine is associated with a greater probability of achieving consistent profound anesthesia. These findings may have practical implications for novice operators during their initial clinical training phase. These associations warrant consideration in undergraduate settings, pending confirmation from randomized controlled trials.

## Figures and Tables

**Table 1:** Baseline demographic characteristics of the patients.

Characteristics	Articaine (*n* = 69)	Lidocaine (*n* = 61)	Mepivacaine (*n* = 59)	*p*-Value
Age, mean (SD)	20.4 (2.1)	20.7 (2.3)	20.5 (2.2)	0.72
Sex, *n* (%)				0.45
Female	36 (52%)	35 (57%)	31 (53%)	
Male	33 (48%)	26 (43%)	28 (47%)	
Tooth position, *n* (%)				0.76
Left third molar	42 (61%)	34 (56%)	29 (49%)	
Right third molar	27 (39%)	27 (44%)	30 (51%)	
Pederson Difficulty, n (%)				0.89
Slightly	23 (33%)	20 (33%)	19 (32%)	
Moderately	46 (67%)	41 (67%)	40 (68%)	

Note: Data are expressed as mean ± standard deviation (SD) for continuous variables and as frequency (*n*) and percentage (%) for categorical variables. Statistical significance for continuous data (age) was evaluated using one-way analysis of variance (ANOVA). For categorical data (sex, tooth position, and difficulty index), Pearson’s chi-square test or Fisher’s exact test was applied as appropriate. A *p*-value < 0.05 was considered statistically significant.

## Data Availability

The data are provided within the manuscript.

## Supplementary Materials

The following supporting information can be downloaded at: https://www.mdpi.com/article/10.3390/dj14030183/s1, Table S1: Distribution of Surgical Cases per Student Operator; Table S2: Distribution of Faculty-Assisted (Excluded) Cases Across Groups (*n* = 24); Table S3: Detailed Analysis of Primary and Secondary Anesthetic Outcomes; Table S4: Mixed-effects Logistic Regression Analysis for Primary Outcome (Stage 2 Success); Table S5: Sensitivity Analysis Including Faculty-Assisted Cases (Total *N* = 213).

## Abbreviations

The following abbreviations are used in this manuscript:

ANOVA	Analysis of variance
HPS	Heft–Parker VAS pain scale
IANB	Inferior alveolar nerve block
IRB	Institutional Review Board
RCT	Randomized controlled trial
STROBE	Strengthening the Reporting of Observational Studies in Epidemiology
