# Supplementary material for: Association of 4% Articaine with Profound Inferior Alveolar Nerve Block Success in Third Molar Surgery Performed by Dental Students: A Three-Anesthetic Observational Study
Source: Dent J (Basel). 2026 Mar 19;14(3):183. doi: 10.3390/dj14030183 (PMC13025740; doi:10.3390/dj14030183)
Supplement: Supplementary file 1 [file dentistry-14-00183-s001.zip › dentistry-4137253-supplementary.pdf]

Article

Articaine Enhances the Success of Profound Inferior Alveolar Nerve Block in Third Molar Surgery Performed by Dental Student: A Three-Anesthetic Observational Study

Thanyaphat Engboonmeskul [1], Rudjit Tunthasen [1], Kannika Rungsaeng [1], Panuwat Rassaiyakarn [1], Poonnapha Tanyacharoen [1], Panuwat Earkun [1] and Teerawat Sukpaita [1,*]

Supplementary Table 1: Distribution of Surgical Cases per Student Operator.

Number of Cases Performed	Number of Operators (n=117)	Total Number of Cases (n=189)	Percentage of Operators (%)
1 Case	50	50	42.70%
2 Cases	62	124	53.00%
3 Cases	5	15	4.30%
Total	117	189	100.00%

Note: The mean number of cases per operator is 1.61 (SD ± 0.57). The distribution reflects the varying number of surgical assignments per student during their clinical rotation. Each operator's involvement was limited to a maximum of three cases to minimize the impact of learning curve bias and ensure a broader representation of novice skill levels.

Supplementary Table 2: Distribution of Faculty-Assisted (Excluded) Cases Across Groups (n=24)

Reasons for Faculty Intervention	Articaine	Lidocaine	Mepivacaine	Total
Failed flap reflection	0	2	1	3
Difficulty in elevating tooth/fragments	2	1	3	6
Failed bone removal or tooth sectioning	5	6	4	15
Total Excluded Cases	7	9	8	24

Note: Cases were excluded from the final analysis if the supervising faculty had to intervene in critical surgical steps. The balanced distribution across the three groups (n=7, 9, 8) suggests that the difficulty of cases was relatively uniform and the anesthetic agent did not disproportionately influence the need for faculty assistance.

Supplementary Table 3: Detailed Analysis of Primary and Secondary Anesthetic Outcomes

Outcome Measures	Articaine (n=69)	Lidocaine (n=61)	Mepivacaine (n=59)	p-value
**Primary Outcome**				
Profound Success Rate (Stage 2), % (n)	76.8% (53)	55.7% (34)	61.0% (36)	0.031*
Components of Stage 2 Success				
Required Supplemental Injection, % (n)	23.2% (16)	44.3% (27)	39.0% (23)	0.024*
Intraoperative Pain >36 mm (HPS), % (n)	2.9% (2)	32.8% (20)	22.0% (13)	<0.001*
**Secondary Outcomes**				
Mean Intraoperative Pain (170 mm HPS)	14.3 ± 10.8	31.0 ± 12.6	29.8 ± 8.8	<0.001*
Anesthetic Onset Time (min)	2.1 ± 0.4	2.2 ± 0.5	2.2 ± 0.4	0.133
Anesthesia Duration (min)	261.7 ± 34.8	164.6 ± 48.3	192.6 ± 33.0	<0.001*
Total Volume of Anesthetic Used (mL)	2.1 ± 0.5	2.4 ± 0.8	2.3 ± 0.8	0.007*

Note: HPS = Heft-Parker Pain Scale (0–170 mm). Stage 2 Success was defined by the absence of supplemental anesthesia and an intraoperative pain score of ≤36 mm (representing 'none to weak pain' per Gurucharan et al., 2022). P-values for secondary outcomes are exploratory and reported without adjustment for multiple comparisons.

Supplementary Table 4: Mixed-effects Logistic Regression Analysis for Primary Outcome (Stage 2 Success).

Variables	Odds Ratio (OR)	95% Confidence Interval (CI)	p-value
**Fixed Effects**			
**Anesthetic Agent**			
* Lidocaine (Reference)	1	-	-
* Articaine	2.41	1.24 – 4.68	0.009*
* Mepivacaine	1.28	0.65 – 2.52	0.472
**Surgical Difficulty (Pederson Scale)**			
* Easy/Moderate (Reference)	1	-	-

* Difficult	0.82	0.41 – 1.64	0.57
**Operator Experience**			
* <5 Prior cases (Reference)	1	-	-
* ≥5 Prior cases	1.15	0.58 – 2.28	0.685
**Random Effects**			
Operator (Variance)	0.12	-	-
Intraclass Correlation (ICC)	0.07	0.01 – 0.28	-

Note: Abbreviations: OR, Odds Ratio; CI, Confidence Interval; ICC, Intraclass Correlation Coefficient; AOR, Adjusted Odds Ratio. *Statistically significant at $p < 0.05$.

Supplementary Table 5: Sensitivity Analysis Including Faculty-Assisted Cases (Total N = 213).

Outcome	Articaine (n=76)	Lidocaine (n=70)	Mepivacaine (n=67)	p-value
Stage 2 Success Rate*	69.7% (53/76)	48.6% (34/70)	53.7% (36/67)	0.021**
Adjusted OR (95% CI)	2.12 (1.12 – 4.01)	1.00 (Ref)	1.18 (0.61 – 2.29)	0.021**

Note: *Faculty-assisted cases (n=24) were all classified as Stage 2 failures.

**Based on mixed-effects logistic regression.
